# Enzymatic Synthesis of Copolyesters with the Heteroaromatic Diol 3,4-Bis(hydroxymethyl)furan and Isomeric Dimethyl Furandicarboxylate Substitutions

**DOI:** 10.1021/acs.biomac.3c01433

**Published:** 2024-04-11

**Authors:** Fitrilia Silvianti, Dina Maniar, Beatriz Agostinho, Tijn C. de Leeuw, Albert Jan Jacob Woortman, Jur van Dijken, Shanmugam Thiyagarajan, Andreia F. Sousa, Katja Loos

**Affiliations:** † Macromolecular Chemistry & New Polymeric Materials, Zernike Institute for Advanced Materials, University of Groningen, Nijenborgh 4, Groningen 9747 AG, The Netherlands; ‡ CICECO—Aveiro Institute of Materials, Department of Chemistry, University of Aveiro, Aveiro 3810-193, Portugal; § CarbExplore Research B.V., Groningen 9747 AA, The Netherlands; ∥ Centre for Mechanical Engineering, Materials and Processes, Department of Chemical Engineering, University of Coimbra Rua Sílvio Lima—Polo II, Coimbra 3030-790, Portugal; ⊥ Wageningen Food & Biobased Research, Wageningen University and Research, P.O. Box 17, Wageningen 6700 AA, The Netherlands

## Abstract

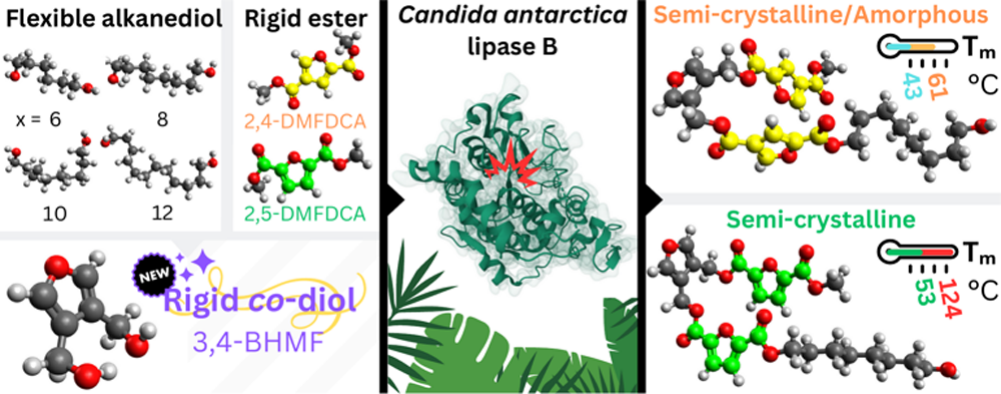

Polyesters from furandicarboxylic acid derivatives, i.e., dimethyl 2,5-furandicarboxylate (2,5-DMFDCA) and 2,4-DMFDCA, show interesting properties among bio-based polymers. Another potential heteroaromatic monomer, 3,4-bis(hydroxymethyl)furan (3,4-BHMF), is often overlooked but holds promise for biopolymer synthesis. Cleaning and greening synthetic procedures, i.e., enzymatic polymerization, offer sustainable pathways. This study explores the *Candida antarctica* lipase B (CALB)-catalyzed copolymerization of 3,4-BHMF with furan dicarboxylate isomers and aliphatic diols. The furanic copolyesters (co-FPEs) with higher polymerization degrees are obtained using 2,4-isomer, indicating CALB’s preference. Material analysis revealed semicrystalline properties in all synthesized 2,5-FDCA-based co-FPEs, with multiple melting temperatures (*T*_m_) from 53 to 124 °C and a glass-transition temperature (*T*_g_) of 9–10 °C. 2,4-FDCA-based co-FPEs showed multiple *T*_m_ from 43 to 61 °C and *T*_g_ of −14 to 12 °C; one of them was amorphous. In addition, all co-FPEs showed a two-step decomposition profile, indicating aliphatic and semiaromatic segments in the polymer chains.

## Introduction

3,4-Bis(hydroxymethyl)furan (3,4-BHMF) was recently discovered as a promising alternative to furanic heteroaromatic diol monomers following the popularity of 2,5-BHMF.^1−4^ Pellis et al.^5^ reported that polyesters derived from 3,4-BHMF exhibited higher thermal stability (*T*_d50%_) than those based on 2,5-BHMF. Typically, 3,4-BHMF is produced through the reduction of dimethyl 3,4-furandicarboxylate (3,4-DMFDCA).^6,7^ The derivation of 3,4-DMFDCA from an agricultural residue through the Henkel reaction involving 2-furoic acid was reported in a previous study.^8^ Additionally, 3,4-DMFDCA was synthesized via a five-step reaction that involved substituted dicarboxylic acid/cyclic anhydride with dimethylmaleic anhydride as the initial reactant.^9^ Notably, both 2-furoic acid and dimethylmaleic anhydride can be obtained from bio-based resources, confirming their renewability.^8,10,11^

2,5-Furandicarboxylic acid (2,5-FDCA) is a key bio-based building block for poly(ethylene furanoate) (PEF) production and has attracted much attention due to its eco-friendly characteristics.^12^ PEF provides a promising future for packaging materials by offering alternatives to traditional fossil-based polyethylene terephthalate.^12−14^ With the prominence of 2,5-FDCA, its heteroaromatic isomer 2,4-FDCA has also recently gained attention, offering increased structural tunability in bio-based polymer synthesis. These two FDCA isomers have different positions in the carboxyl groups, which influences their reactivity and intramolecular interactions within the polymer chain, including the ability of the polymer chains to fold and crystallize. FDCA-based polymers based on these two isomers were reported to exhibit different characteristics and properties.^15−18^ As highlighted in several reports, in comparison to 2,5-FDCA, the 2,4-FDCA isomer has been observed to disrupt ordering and crystallization in the polymer chain.^15,18,19^

Numerous requirements have been introduced in the pursuit of developing sustainable polymers. In recent decades, there has been a dramatic increase in the use of sustainable polymers derived from bio-based raw materials.^20,21^ However, questions have also been raised about the sustainability of the synthesis procedure, e.g., the catalysts and energy consumption. Enzymes have emerged as promising options for renewable catalysts because they originate from bio-based resources, thereby providing a clean process that ensures that no detrimental byproducts contaminate the resulting product or the environment.^22−26^ Furthermore, enzymes catalyze reactions under mild conditions at lower temperatures than metal catalysts.^20,22−24^ In addition, several studies have shown that enzymes have high selectivity.^20,25−28^ For example, *Candida antarctica* Lipase B (CALB), the most extensively studied enzyme in polyester synthesis, showed regioselectivity toward primary alcohol in polymerization utilizing polyols, such as glycerol.^29,30^ CALB selectivity has also been reported to be applicable for isomeric structures, and a higher conversion rate was achieved when polymerizing isomannide with succinic acid than when using isosorbide to synthesize linear and cyclic ester oligomers.^31^ In our previous work,^32^ CALB showed a preference for 2,5-FDCA dimethyl esters over the 2,4-isomer in furan-based polyester synthesis.

This study aims to fill the knowledge gap on the enzymatic polymerization of furan heteroaromatic diol 3,4-BHMF. In order to render polymer with enhanced rigidity, 3,4-BHMF was enzymatically polymerized in combination with furan-based esters and aliphatic diols. CALB preferences for catalyzing 3,4-BHMF with different furanic isomers (2,5- or 2,4-FDCA dimethyl ester) and various aliphatic diols with different chain lengths (6, 8, 10, and 12 methylene units) were evaluated. Finally, the properties of the resulting polymer derived from the furan heteroaromatic diol 3,4-BHMF were studied.

## Materials and Methods

### Materials

Novozym 435 (N435, *Candida antartica* lipase B (CALB)) immobilized on acrylic resin (5000+ U g^–^^1^), 1,6-hexanediol (1,6-HDO, 99%), 1,8-octanediol (1,8-ODO, 98%), 1,10-decanediol (1,10-DDO, 98%), 1,12-dodecanediol (1,12-DODO, 99%), chloroform (CHCl_3_, Chromasolv HPLC, ≥99.8%, amylene stabilized), deuterated chloroform (CDCl_3_, 99.8 atom % D), potassium trifluoroacetate (KTFA, 98%), and 3,4-bis(hydroxymethyl)furan (3,4-BHMF, 98%) were purchased from Sigma-Aldrich. Toluene (anhydrous, 99.8%) was purchased from Alfa Aesar. Dimethyl 2,5-furandicarboxylate (2,5-DMFDCA, 97%) was purchased from Fluorochem UK. Absolute methanol (MeOH, AR grade) was obtained from Biosolve Chemicals. 1,1,1,3,3,3-Hexafluoro-2-propanol (HFIP, ≥99%) was acquired from TCI Europe. Dithranol (≥98%) was purchased from Fluka.

N435 was predried, as reported previously.^33^ Dimethyl 2,4-furandicarboxylate (2,4-DMFDCA) was synthesized using a previously described procedure.^8^ The molecular sieves (4 Å) were preactivated at 200 °C. All of the other chemicals were used as received.

### One-Step iCALB-Catalyzed Polycondensation

As detailed below, we employed enzymatic polymerization procedures to synthesize furan-based copolyesters (co-FPEs), which were constructed upon previous research.^4^ Our experimental approach involved copolymerizing DMFDCA, 3,4-BHMF, and an aliphatic diol using a fixed molar ratio. In particular, we employed a DMFDCA/3,4-BHMF/aliphatic diol feed molar ratio of 50:12.5:37.5, with the following procedure. First, a predried N435 and preactivated molecular sieve (15 and 150 wt % of the monomer, respectively) were introduced into a 25-mL round bottle under a nitrogen atmosphere. Subsequently, DMFDCA (265.4 mg, 1.44 mmol), 3,4-BHMF (46 mg, 0.36 mmol), 1,10-DDO (188.4 mg, 1.08 mmol), and anhydrous toluene (2.5 mL) were added to the flask. The flask was then immersed in a preheated oil bath at 90 °C and subjected to magnetic stirring under a nitrogen atmosphere. After 72 h, the reaction was allowed to cool to room temperature. Chloroform (±10 mL) was added to the mixture, and the mixture was vigorously stirred to dissolve the polymer products. N435 and the molecular sieves were filtered using standard filtering techniques (folded filter type 15 Munktell 240 mm) and then washed with chloroform (2 × 10 mL). All of the obtained solutions were mixed and concentrated using a rotary evaporator set to 40 °C under a reduced pressure of 400–480 mbar. Precipitation of the concentrated solution in excess cold methanol was performed. The precipitated product was then recovered using centrifugation (5 min, 4500 rpm, 4 °C) in a Thermo/Heraeus Labofuge 400 R and dried for ±3 days under vacuum at 40 °C. Finally, the products were kept at room temperature before analysis.

### Procedure for the Two-Step iCALB-Catalyzed Polycondensation

The initial 2 h reaction was carried out at 90 °C under a nitrogen atmosphere with continuous magnetic stirring for the first step. A second step was subsequently conducted under vacuum at 600 mmHg for 70 h. The procedure for polymerization and the composition of the monomers, DMFDCA, 3,4-BHMF, and aliphatic diol, were determined in a manner similar to the previously mentioned one-step approach.

### Analytics

Proton nuclear magnetic resonance (^1^H NMR; 600 MHz) spectra were recorded on a Bruker Ascend NMR600 spectrometer using CDCl_3_ as the solvent.

Attenuated total reflection-Fourier transform infrared (ATR-FTIR) spectra were recorded on a Bruker VERTEX 70 spectrometer equipped with an ATR diamond single reflection accessory. The measurement resolution was 4 cm^–1^, and the spectra were collected in the range 4000–400 cm^–1^, with 16 scans for each sample. Atmospheric compensation and baseline correction were applied to the collected spectra using OPUS spectroscopy software (v7.0) (Bruker Optics).

The molecular weights (number-average, *M*_n_, and weight-average, *M*_w_) of the co-FPEs were determined via a size exclusion chromatography (SEC) instrument equipped with a triple detector, consisting of a Malvern Dual detector and Schambeck RI2012, a refractive index detector. Separation was carried out by utilizing two PLgel 5 μm MIXED-C 300 mm columns from Agilent Technologies at 35 °C. HPLC grade chloroform was used as the eluent with a flow rate of 0.5 mL min^–1^. Data acquisition and calculations were performed using Viscotek OmniSec software version 5.0. Molecular weights were determined based on a conventional calibration curve generated from narrow dispersity polystyrene standards (Agilent and Polymer Laboratories, *M*_w_ = 645–3,001,000 g mol^–1^). The samples were filtered through a 0.2 μm PTFE filter prior to injection.

Matrix-assisted laser desorption/ionization-time-of-flight mass spectrometry (MALDI-ToF MS) was performed using a 4800 Plus MALDI TOF/TOF Analyzer (Applied Biosystems) in reflector positive mode. The matrix, cationization agent, and solvent used were dithranol, KTFA, and HFIP, respectively. First, dithranol (20 mg mL^–1^), KTFA (5 mg/mL) and a polymer sample (1–2 mg mL^–1^) were premixed at a ratio of 5:1:5. The mixture was subsequently hand-spotted on a stainless steel plate and left to dry. The presence of copolyesters with different end groups was determined by the following equation:
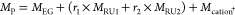
1where *M*_P_ is the molecular mass of a copolyester species, *M*_EG_ is the molecular mass of the end groups, *r*_1_ and *r*_2_ are the numbers of each segment, *M*_RU_ is the molecular mass of the segment, and *M*_cation^+^_ is the molecular mass of the potassium cation, sodium cation, or hydrogen cation.

The analysis of the thermal properties was performed on a TA-Instruments Q1000 DSC instrument, which was calibrated with indium as the standard. The heating rate was 10 °C min^–1^ under a nitrogen flow. The product melting points (*T*_m_) were measured by a second heating scan. The glass transition temperatures (*T*_g_) were measured by a second heating scan or temperature-modulated differential scanning calorimetry (TMDSC) at 2 °C min^–1^ with a temperature modulation of ±0.50 °C every 60 s.

The thermal stability and degradation temperature were analyzed via thermogravimetric analysis (TGA) on a TA-Instruments Discovery TGA 5500 instrument using a heating rate of 10 °C min^–1^ in a nitrogen environment.

Wide-angle X-ray diffraction (WAXD) patterns were recorded on a Bruker D8 Endeavor diffractometer with Cu Kα radiation (λ = 0.1542 nm) in the angular range of 5–50° (2θ) at room temperature. The percentage of crystallinity (χ_c_) was determined using the WAXD spectra through the following formula: χ_c_ (%) = 100 × *k* × *I*_c_/(*I*_c_ + *I*_a_).^1,34^ Here, *k* represents the relative scattering factor between a polymer’s crystal and amorphous parts (typically assumed as *k* = 1 due to its difficulty of determination). The integration intensities of the crystal peaks (*I*_c_) and the integration intensity of the amorphous halo (*I*_a_) were obtained by using the Peak Analyzer tool available in OriginPro 9.1 software by OriginLab Corporation.

## Results and Discussion

### Synthesis and Structural Characterization of Co-FPEs

**Scheme 1:**
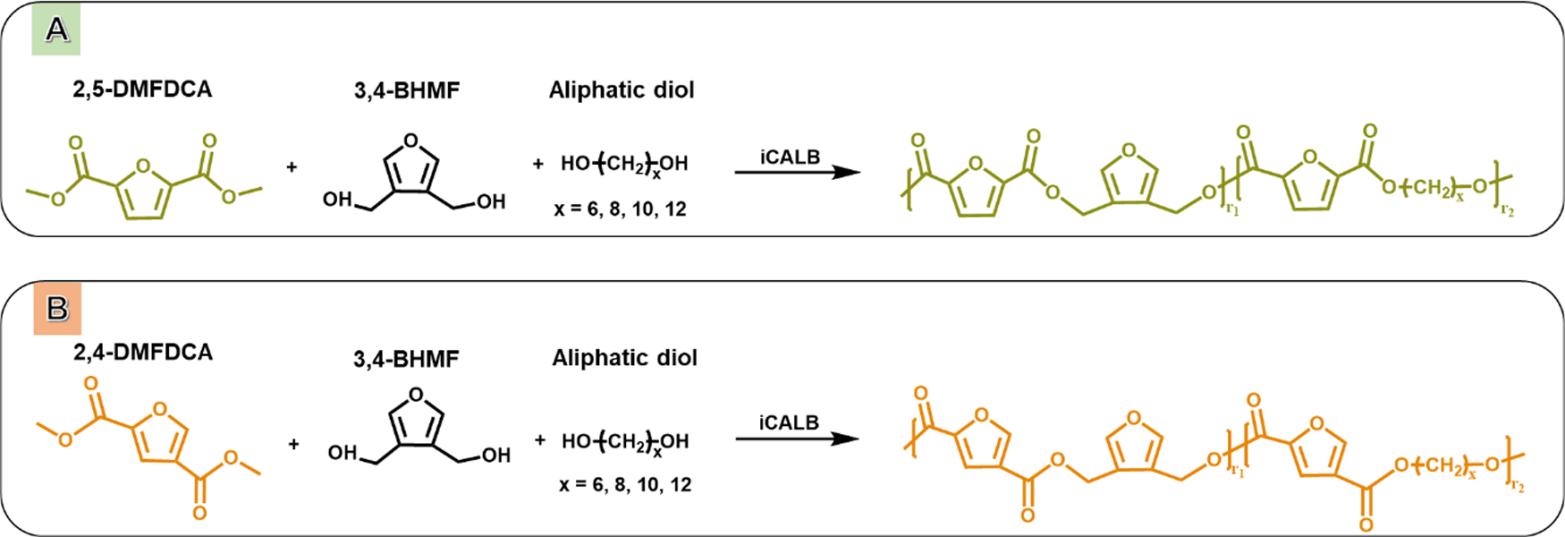
Enzymatic Polymerization of Co-FPEs from (a) 2,5- and (b) 2,4-DMFDCA in Combination with 3,4-BHMF and Linear Aliphatic Diols

co-FPEs were synthesized via enzymatic polycondensations, as outlined in Scheme 1. The CALB-catalyzed reaction was performed using two isomeric DMFDCA monomers, namely, 2,5- and 2,4-DMFDCA. A uniform approach was adopted for the synthesis of co-FPEs to assess the reactivity of these two FDCA dimethyl ester (DMFDCA) isomers in enzymatic polymerization. In accordance with our prior research on the enzymatic synthesis of furan-based polyesters,^34^ the use of DMFDCA is favored over FDCA due to improved solubility of the former. The use of the heteroaromatic furan diol 3,4-BHMF was introduced in this work. 3,4-BHMF has been previously reported to improve the thermal stability of polyesters synthesized with dimethyl succinate (DMS), dimethyl adipate (DMA), and dimethyl sebacate (DMSe).^5^ In this work, the aim was to explore the potential of enhancing the rigidity of polymer chain structures by incorporating 3,4-BHMF in combination with specified furan-based isomeric esters and aliphatic diols. The aliphatic linear diols employed in the synthesis feature varying chain lengths comprising 6, 8, 10, and 12 methylene units (*x*). The synthesized co-FPEs are outlined in Table 1.

**Table 1:** Abbreviations for the synthesized co-FPEs

*x*^a^	DMFDCA isomer	copolyester	abbreviation
6	2,5-DMFDCA	poly(3,4-furandimethylene furanoate-*co*-hexamethylene-2,5-furanoate)	P(3,4-FMF-*co*-2,5-HF)
8		poly(3,4-furandimethylene furanoate-*co*-octamethylene-2,5-furanoate)	P(3,4-FMF-*co*-2,5-OF)
10		poly(3,4-furandimethylene furanoate-*co*-decamethylene-2,5-furanoate)	P(3,4-FMF-*co*-2,5-DF)
12		poly(3,4-furandimethylene furanoate-*co*-dodecamethylene-2,5-furanoate)	P(3,4-FMF-*co*-2,5-DOF)
6	2,4-DMFDCA	poly(3,4-furandimethylene furanoate-*co*-hexamethylene-2,4-furanoate)	P(3,4-FMF-*co*-2,4-HF)
8		poly(3,4-furandimethylene furanoate-*co*-octamethylene-2,4-furanoate)	P(3,4-FMF-*co*-2,4-OF)
10		poly(3,4-furandimethylene furanoate-*co*-decamethylene-2,4-furanoate)	P(3,4-FMF-*co*-2,4-DF)
12		poly(3,4-furandimethylene furanoate-*co*-dodecamethylene-2,4-furanoate)	P(3,4-FMF-*co*-2,4-DOF)

a The number of methylene units in aliphatic linear diols.

**Figure 1:**
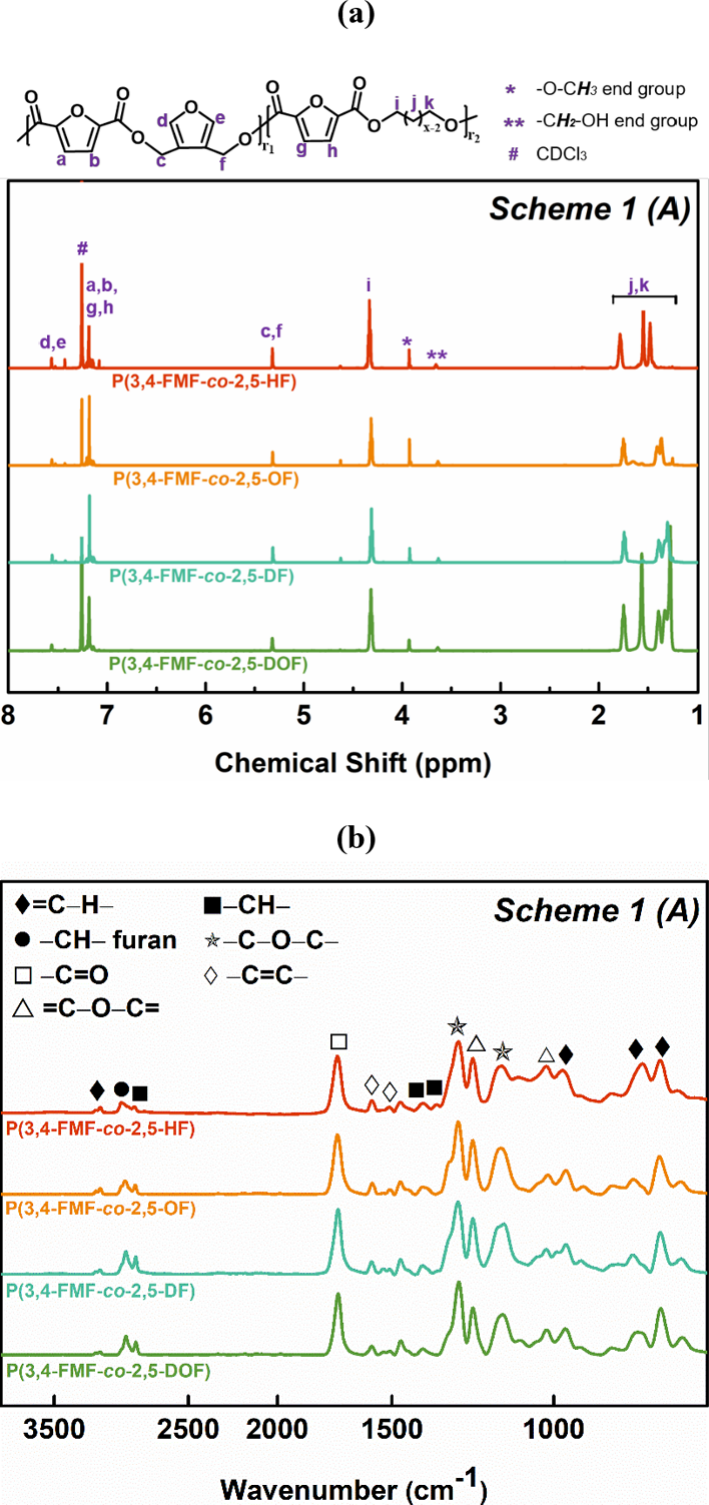
(a) ^1^H NMR and (b) ATR-FTIR spectra of co-FPEs obtained from the enzymatic polymerization of 3,4-BHMF, 2,5-DMFDCA, and aliphatic diols.

The successful synthesis of the co-FPEs was confirmed by ^1^H NMR and ATR-FTIR spectroscopy, as shown in Figures 1 and S1, respectively. The presence of signals at approximately 4.27–4.33 and 5.24–5.32 ppm, corresponding to −C(O)O–C*H*_2_– (i) and −C*H*_2_– (f), respectively, confirms the formation of ester bonds from each co-FPE isomer in Scheme 1 (see Figures 1a and S1a). Two identical triplet peaks at approximately 4.25 and 4.30 ppm and four singlet peaks corresponding to asymmetric 2,4-DMFDCA were observed in the ^1^H NMR spectra of 2,4-*co*-FPEs synthesized from the reaction in Scheme 1B (see Figure S1a).^18^ Furthermore, the asymmetry of 2,4-DMFDCA is reflected by the appearance of two singlet furan signals at 8.08 and 7.44 ppm and two singlet protons from the −O–C*H*_3_ end groups at 3.86 and 3.91 ppm. However, only one furan proton peak at 7.18 ppm was observed in the symmetrical furan structures of 2,5-co-FPEs (Figure 1a).

Furthermore, the ATR-FTIR spectra in Scheme 1A,B provide additional evidence of ester linkage formation for both reactions through the sharp band at approximately 1721 cm^–1^, corresponding to the C=O stretching vibration of the ester groups consistent with previous research on furanic polymers,^4,5,32^ to represent the ester vibration from furan monomer. Figures 1b and S1b display absorption bands related to the furan heterocycle, including a weak band at 3118–3137 cm^–1^ arising from the =C–H stretching vibration of the furan ring and bands at 1573–1583 and 1506–1511 cm^–1^ assigned to the aromatic C=C bending vibrations.^4,34^ In accordance with the findings of previous studies, the variations between furan isomers are shown through peak shifts in the asymmetrical and symmetrical stretching vibrations of the C=C group, the ring vibration of the furan ring of =C–O–C= , and the out-of-plane deformation vibration of the furan rings of =C–H.^18,19^ More detailed information about the assignments of the ^1^H NMR and FTIR peaks can be found in the Materials and Methods section.

**Figure 2:**
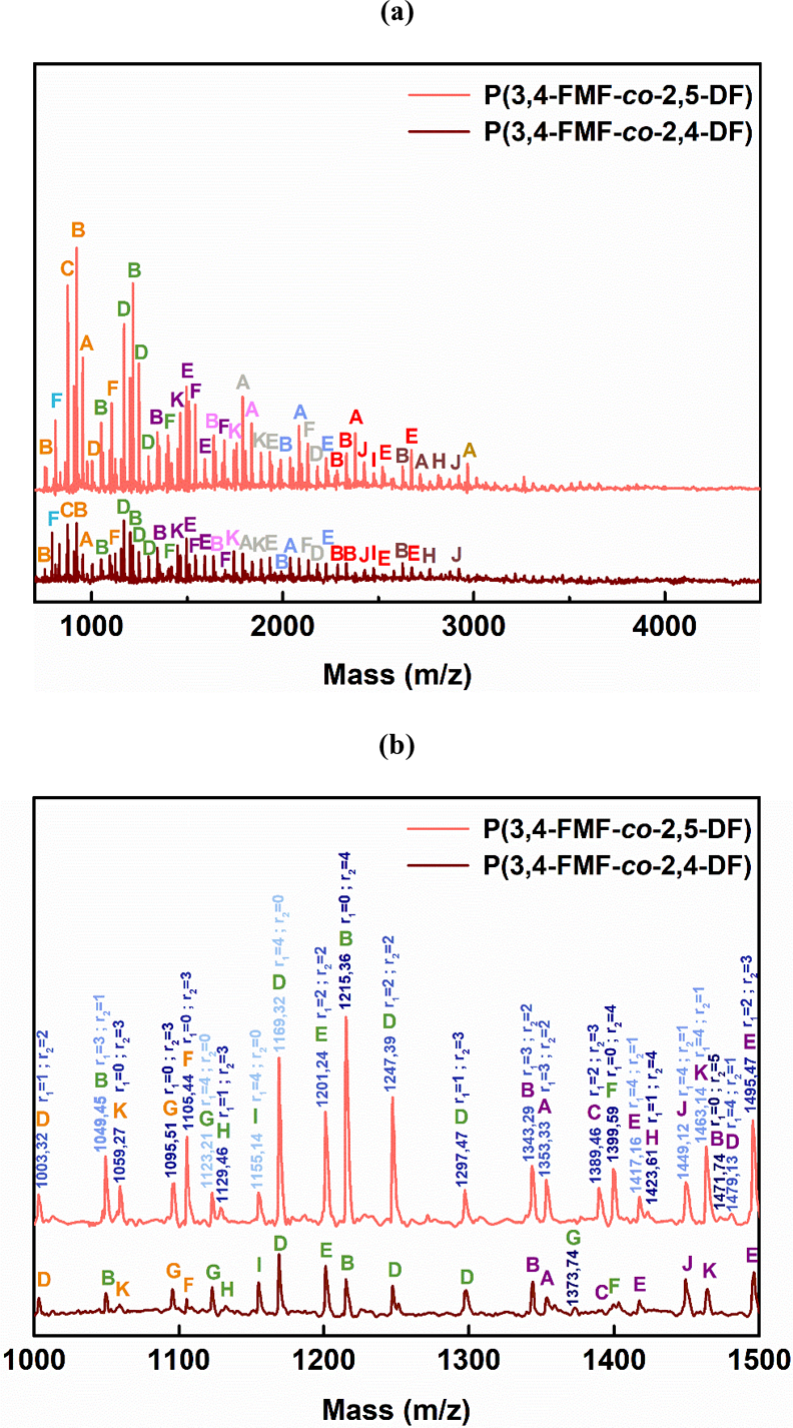
(a) MALDI-ToF MS spectra of co-FPEs synthesized from 3,4-BHMF, 1,10-decanediol, and two different DMFDCA isomers (2,5- and 2,4-DMFDCA), and (b) magnified spectra.

MALDI-ToF MS was employed to further investigate the microstructures and end groups of the obtained co-FPEs. The representative mass spectra of co-FPEs are shown in Figure 2, covering the *m*/*z* range from 700 to 5000. Distinct peak separations (*m*/*z*) were observed in the P(3,4-FMF-*co*-2,5-DF) and P(3,4-FMF-*co*-2,4-DF) MALDI-ToF MS spectra. Two different molecular weights corresponding to the aliphatic and heteroaromatic repeating units were observed, with values of 294.35 and 263.23 g mol^–1^, respectively. A similar pattern was observed in the MALDI-ToF MS spectra of all tested co-FPEs, revealing the presence of 11 distinct species, as summarized in Table 2. Both P(3,4-FMF-*co*-2,5-DF) and P(3,4-FMF-*co*-2,4-DF) exhibited the same dominant peaks as linear species A and cyclic species B. The present findings seem to be consistent with other research on furan isomer polyesters synthesized via CALB-catalyzed polymerization.^34−37^

**Table 2:**
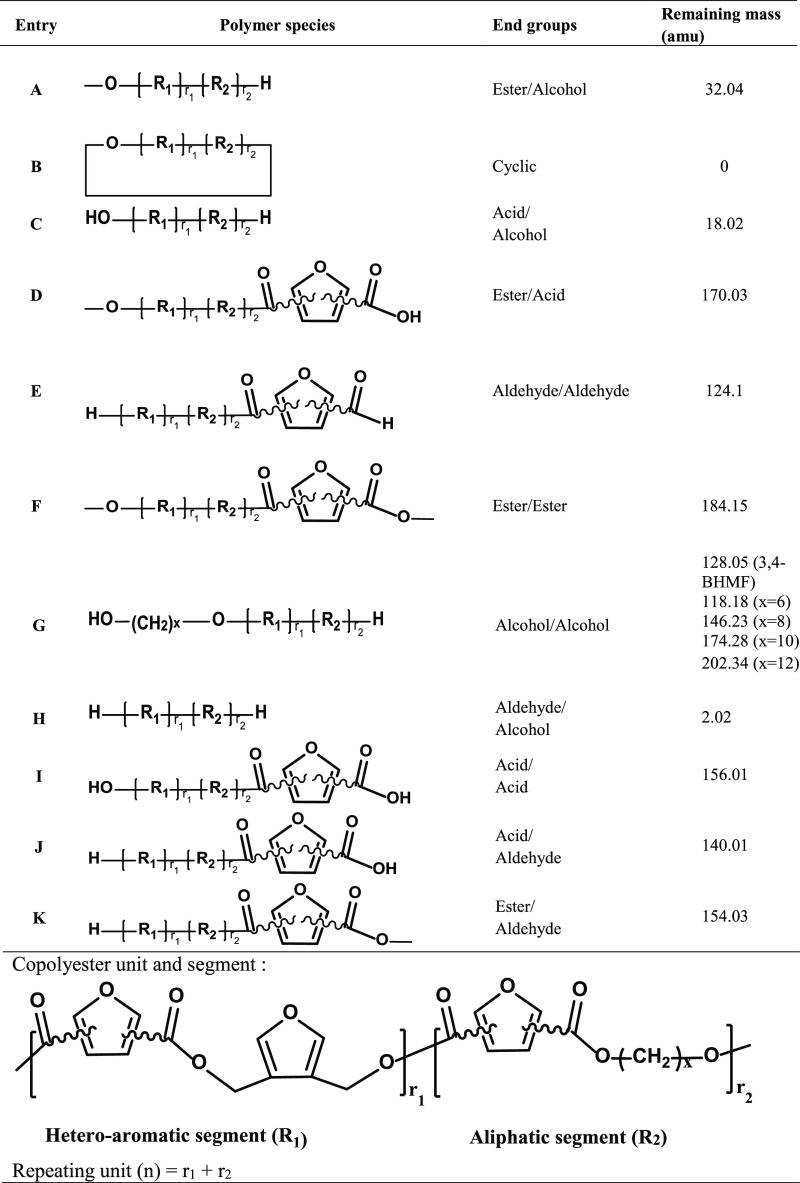
MALDI-ToF MS Analysis: End Groups of the Obtained Copolyesters

Additionally, we can see from the MALDI-ToF MS results that the prediction of *m*/*z* based on the possible variation in repeating units would indicate that the co-FPEs are classified as random copolymers. As displayed in Figure 2, species with the same color represent similar repeating unit numbers (*n*). In addition, an increasing number of aliphatic units (*r*_1_) is represented by an increasing blue gradient, while in contrast, the number of heteroaromatic units (*r*_2_) decreases (see Figure 2b). This random polymer arrangement generally occurs when the three monomers are polymerized together through transesterification.^38,39^

### Influence of Isomeric Substitution and Aliphatic Diol Chain Length on the Enzymatic Synthesis of Co-FPEs

**Figure 3:**
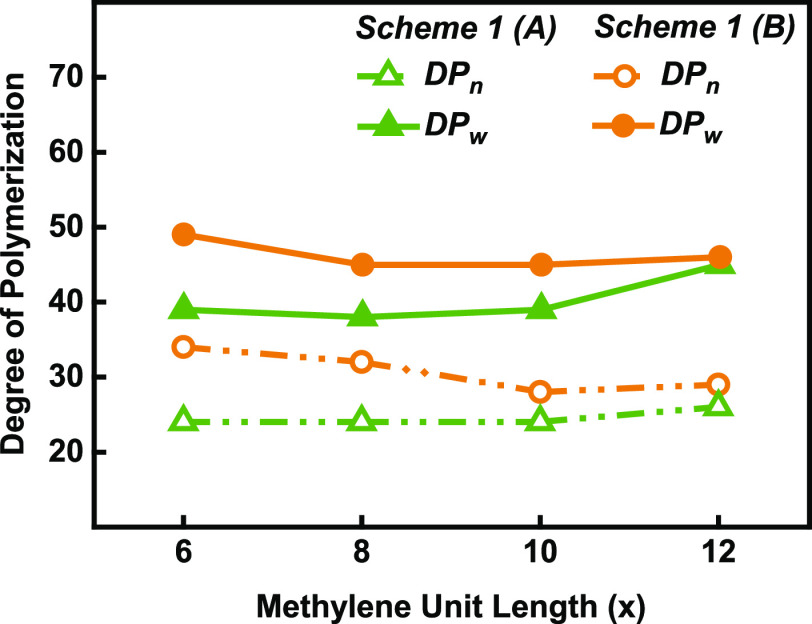
DP_*n*_ and DP_*w*_ of the co-FPEs from the two routes in Scheme 1 involving 2,5- or 2,4-FDCA dimethyl esters, 3,4-BHMF, and various aliphatic diols against the chain length of the aliphatic monomer.

The effect of the isomeric substitution of 2,5- and 2,4-DMFDCA isomers on the enzymatic synthesis of co-FPEs with aliphatic diol as the flexible spacer unit and 3,4-BHMF as a heteroaromatic comonomer was examined. Molecular weights from SEC analysis, yield, and dispersities of the obtained co-FPEs are summarized in Table S1. The degree of polymerization (DP) was calculated using the same method, as reported previously.^4^ The DP is plotted against the length of the methylene unit of the aliphatic diols in Figure 3. The results indicate that CALB prefers to copolymerize asymmetrical 2,4-DMFDCA isomers over symmetrical 2,5-DMFDCA isomers. This preference is evidenced by higher values of the number-average degree of polymerization (DP_*n*_) and weight–average degree of polymerization (DP_*w*_). As highlighted in previous work,^32^ the asymmetrical structure of 2,4-DMFDCA creates a unique arrangement that influences its interaction with the enzyme’s active site. The methyl ester groups of 2,4-DMFDCA are oriented toward the hydrophobic wall of amino acids in the CALB active site cleft, freeing up space for a heteroaromatic or aliphatic diol to attack the acyl intermediate. Therefore, this approach reduces steric hindrance during copolymerization with a heteroaromatic furan diol and an aliphatic diol. In contrast, the reaction depicted in Scheme 1A involves a symmetrical furanic isomer, 2,5-DMFDCA, which tends to cluster carboxyl groups, as observed in our previous work.^32^ In this case, the 2,5-DMFDCA methyl groups are oriented away from the enzyme wall, resulting in steric hindrance that restricts the propagation of the polymer chain.

The results from the reaction depicted in Scheme 1A provide noteworthy insights into the effects of the aliphatic comonomer as a spacer on enzymatic polymerization. We observed relatively consistent values for DP_*n*_, approximately 24 for aliphatic unit chain lengths of 6–10. Similarly, the DP_*w*_ values are relatively constant at approximately 38 and 39. However, increases in DP_*n*_ and DP_*w*_ occur when copolymerizing with *x* = 12 diol to 26 and 45, respectively. These observed trends agree with previous studies and appear to support the discussion in the previous section regarding the influence of monomer orientation on steric hindrance during copolymerization.^40^ The role of the aliphatic diol as an aliphatic bridge between heteroaromatic monomers becomes evident in this context.^41,42^ Our previous work, while preliminary, suggested that the enzymatic polycondensation of 100% furan diols and furan diester results in only a low-molecular-weight product (see Figure S5). In this work, the incorporation of 75% aliphatic diol effectively mitigated steric hindrance and facilitated copolymer formation. The ^1^H NMR data corroborate a negative correlation between rigid diol incorporation and DP: polymers with a lower DP_*n*_ in Figure 3 have a higher mol % 3,4-BHMF in the isolated polymer (Figure S6).

Among the synthesized asymmetrical 2,4-FDCA-based co-FPEs (see Scheme 1B), P(3,4-FMF-*co*-2,4-HF) (*x* = 6) exhibited the highest numbers for both DP_*n*_ and DP_*w*_, reaching 34 and 49, respectively. Like in the synthesis of 2,5-FDCA-based co-FPEs, variations in the aliphatic diol length (*x* = 6 to *x* = 12) did not significantly affect the DP_*n*_ or DP_*w*_. For instance, at *x* = 8 and *x* = 10, DP_*w*_ remained constant at 45, while their respective DP_*n*_ values were 32 and 28. The longest diol chain length (*x* = 12) displayed a relatively similar value, with a DP_*n*_ of 29 and DP_*w*_ of 46. In contrast to the trends observed for 2,5-FDCA-based co-FPEs, a preference for a shorter aliphatic diol length (*x* = 6) was observed. This rather contradictory result may be explained by the cyclization reaction. Previous studies have reported that during step-growth polymerization, the creation of linear products can be impeded by simultaneous cyclization reactions.^35,36,43,44^ To gain a more comprehensive understanding of the trends in DPs, we further investigated the MALDI-ToF MS results and quantified the relative abundance of each polymer species from the peak intensities, as illustrated in Figure S2. However, despite the advantages of MALDI-ToF MS for providing direct identification of polymer product species, it is essential to note that we should approach this analysis with caution because end groups frequently influence the ionization efficiency of MALDI-TOF MS.^45−49^ Polymer chains may vary in their readiness for ionization based on their chain ends, potentially leading to an overestimation of certain end groups compared to others. Based on the results in Figure S2, it becomes apparent that the 2,5-DMFDCA-based co-FPEs exhibited a greater incidence of cyclic species (B) than the 2,4-DMFDCA-based co-FPEs. This difference may be attributed to the use of symmetrical 2,5-DMFDCA and 3,4-BHMF as comonomers. It is well-known that CALB prefers to form cyclic polymers when reacting with symmetrical structures rather than asymmetrical structures.^36^ As observed in the MALDI-ToF MS spectrum of P(3,4-FMF-*co*-2,5-DOF), the highest fraction of the total peak intensity, 41%, was attributed to cyclic species (B). Hence, it is reasonable to conclude that the DP trends in 2,5-FDCA-based co-FPEs are mainly attributed to cyclic rather than linear species. However, in 2,4-FDCA-based co-FPEs, a less pronounced disparity in the number of cyclic fractions was observed in the MALDI-ToF MS spectra, ranging from 10 to 15%. It can thus be suggested that the use of symmetrical 3,4-BHMF and asymmetrical 2,4-DMFDCA can change the chain and functional group orientations within the enzyme’s active site, thereby limiting the formation of cyclic species. However, it is important to acknowledge that other factors can influence enzymatic catalysis efficiency, including the role of aliphatic diols as comonomers, dilution conditions, enzyme specificity toward substrates, monomer feed concentrations, viscosity, temperature, and the effectiveness of side product removal.^35−37,44,50^ In the following section, we study the use of a two-step polymerization approach to improve side product removal under vacuum conditions.

### Influence of the Copolymerization Method

**Figure 4:**
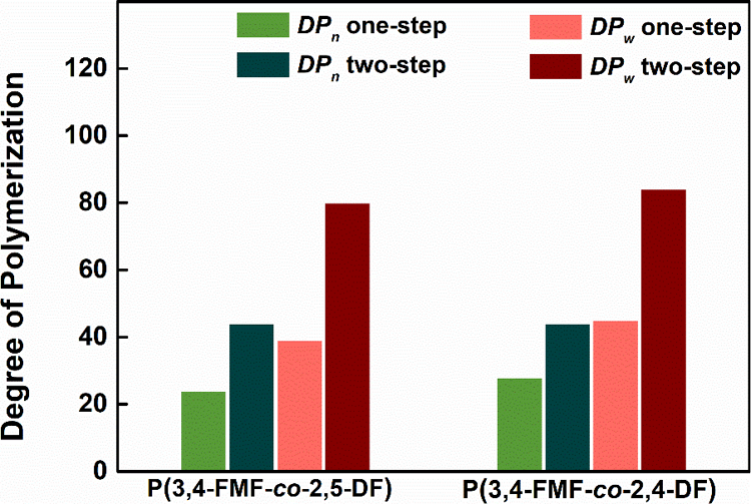
Comparison of DP_*n*_ and DP_*w*_ results of P(3,4-FMF-*co*-2,5-DF) and P(3,4-FMF-*co*-2,4-DF) in one- and two-step methods.

Numerous studies provide valuable insights into preparing high-molecular-weight polymers via polycondensations. Precise reaction conditions are vital for obtaining a high-molecular-weight product by counteracting stoichiometric imbalances due to side reactions and monomer evaporation. Controlling the temperature during the initial stage of polymerization mitigated the monomer volatilization. Subsequently, side product elimination is facilitated through vacuum treatment in the second step.^21,51^ In this work, methanol was generated as a side product of the transesterification reaction. Therefore, to improve the removal of methanol, a two-step method with 600 mmHg vacuum after 2 h of precondensation was used to synthesize P(3,4-FMF-*co*-2,5-DF) and P(3,4-FMF-*co*-2,4-DF). Figure 4 illustrates DP_*n*_ and DP_*w*_ improvement through a two-step method. Both P(3,4-FMF-*co*-2,5-DF) and P(3,4-FMF-*co*-2,4-DF) exhibited similar trends in terms of molecular weight improvement. P(3,4-FMF-*co*-2,5-DF) exhibited DP_*n*_ and DP_*w*_ enhancements from 24 to 44 and from 39 to 80, respectively. Similarly, 3,4-FMF-*co*-2,4-DF shows increases in DP_*n*_ and DP_*w*_ of 58 and 85%, respectively.

In comparison to co-FPEs obtained from the one-step method, two-step polycondensations reveal differences in the compositions of the polymer species (Figure S3). In P(3,4-FMF-*co*-2,5-DF), the number of cyclic species (B) increased substantially by 90%, and there was a 68% increase in the number of linear species with acid/aldehyde end groups (J). However, species A, C, and H displayed decreasing trends, suggesting increased cyclic species formation by backbiting due to carboxyl and hydroxyl end groups.^35−37^ These findings agree with our earlier observations,^32^ which showed that CALB prefers cyclic product formation in symmetrical furan-based polyesters. However, in P(3,4-FMF-*co*-2,4-DF), cyclic species showed only a 19% increase, and linear species G and D showed remarkable increases of 291 and 120%, respectively. These results indicate that by using a two-step polymerization reaction the cyclization reaction is more apparent when using a symmetrical DMFDCA isomer. However, in P(3,4-FMF-*co*-2,4-DF), where a nonsymmetric DMFDCA isomer is employed, the improvement in the number of linear chains is more apparent. This result agrees with the findings of another study of dimethyl terephthalate-based polyesters; due to the unrestricted rotation of hydroxy groups on the symmetrical structure, the formation of enzyme–acyl intermediates is promoted.^36^ In addition, implementing a two-step process facilitates more efficient cyclization by minimizing interference from byproducts.^52^ However, according to existing studies, another factor, specifically reaction temperature, appears to be more influential in promoting the formation of cyclic products.^37,53−56^ To definitively determine the crucial factors involved in this work, further investigation is needed as we did not vary the temperature.

### Thermal Properties of the Obtained co-FPEs

As shown in Figure S4 and Table S2, all of the copolyesters exhibited a two-step degradation profile in which the maximum decomposition rate (*T*_d-max_) occurred in the first step (*T*_d-max-1_), ranging from approximately 389 to 400 °C, and in the second step (*T*_d-max-2_), ranging from approximately 467–501 °C. This finding suggested that the copolyester comprises two distinct segments. Since the monomer feed contains a lower amount of heteroaromatic diol than aliphatic diol and considering the greater weight loss during the initial step as opposed to the second step, we can infer that the degradation temperature of the first step corresponds to the aliphatic segment (R_2_; see Table 2) of the copolyester. These findings align with previous research^26,34^ on aliphatic furan polyesters in which *T*_d-max_ was within the same range as for *T*_d-max-1_ in this work. In contrast, the second step *T*_d-max-2_ is associated with the heteroaromatic segment (R_1_; Table 2), which is in agreement with previous work on 3,4-BHMF-based polyesters.^5^ Moreover, our investigation revealed that 2,4-FDCA-based co-FPEs (see Scheme 1B) possess a higher *T*_d-max_ than 2,5-FDCA-based co-FPEs (see Scheme 1A), which agrees with the difference in molecular weight. This observation also aligns with previous studies on the 2,4-DMFDCA isomer, which showed that 2,5-FDCA-based polymers exhibit higher *T*_d-max_ values than 2,4-isomer products.^15,18^ In addition, as previously reported for poly(butylene 2,4-furanoate) (2,4-PBF), in comparison to 2,5-PBF, a higher aromaticity could also contribute to a higher degradation temperature.^15^

Interestingly, the findings indicate that through variations in the diol chain length there is no substantial difference in *T*_d-max-1_, but a notable distinction emerges in *T*_d-max-2_. This can be attributed to the predominance of the heteroaromatic segment (R_1_; see Table 2), which could be more prevalent in the polymer product, leading to an elevated *T*_d-max-2_. In particular, in 2,5-FDCA-based co-FPEs, P(3,4-FMF-*co*-2,5-HF) (*C* = 6) demonstrated the highest *T*_d-max-2_ at 501 °C (see Figure S4a). However, for 2,4-FDCA-based co-FPEs, P(3,4-FMF-*co*-2,4-OF) (*C* = 8) had the highest *T*_d-max-2_ at 480 °C. This indicates that these polymers contain the highest number of heteroaromatic segments (R_1_; see Table 2). In addition, it is notable that the overall *T*_d-max_ profiles of the copolyesters in this work, which utilized 3,4-BHMF as a heteroaromatic diol, were greater than those of the copolyesters with 2,5-BHMF.^4^ This finding also agrees well with previous work by Pellis et al.,^5^ which showed that 3,4-BHMF-based polyesters exhibit greater *T*_d-max_ values than 2,5-BHMF.

**Figure 5:**
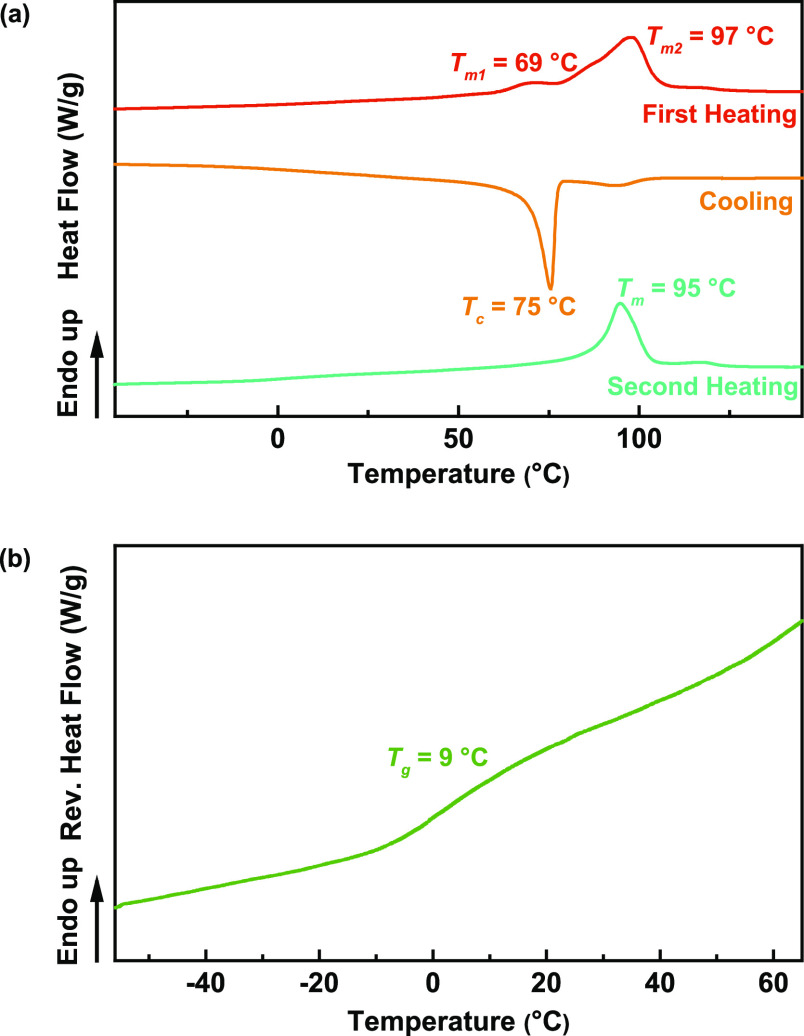
(a) DSC curves and (b) TMDSC curves of P(3,4-FMF-*co*-2,5-DF).

In addition to TGA, we conducted a comprehensive thermal analysis of the co-FPE series using DSC. The thermal characteristics of all co-FPEs are summarized in Table S2. Figure 5 shows representative DSC and TMDSC profiles for P(3,4-FMF-*co*-2,5-DF). The 2,5-FDCA-based co-FPEs exhibited two broad melting peaks in the first heating and a crystallization peak in the cooling scan. All of the obtained 2,5-FDCA-based co-FPE materials exhibited similar glass transition temperatures (*T*_g_) of approximately 9 and 10 °C, regardless of the aliphatic diol chain length. This may be attributed to the more apparent effect of the relative quantity of heteroaromatic components in these co-FPEs. P(3,4-FMF-*co*-2,5-HF) (*C* = 6) had the lowest molecular weight and the highest *T*_d-max-2_, indicating the presence of a high heteroaromatic segment in this product. This underscores the role of incorporating rigid components into the polymer’s main chain, a well-known strategy for enhancing thermal stability.^57−59^ On the other hand, a consistent decrease in the melting temperature (*T*_m_) was observed as the chain length of the aliphatic linear diol increased. This decrease in *T*_m_ can be attributed to the increasing chain flexibility and enhanced mobility resulting from longer aliphatic chains.^4^ This decreasing trend in the *T*_m_ aligns well with earlier conclusions drawn from studies on furan-based polyesters and copolyesters.^4,34^ 2,4-FDCA-based co-FPEs exhibit diverse DSC profiles. Except for P(3,4-FMF-*co*-2,4-DOF), no *T*_m_ or crystallization peak was evident in the second heating scan. For co-FPEs with *x* = 8–12, we observed a decreasing trend in *T*_g_ below 0 °C, correlating with an increase in the length of the methylene chain. This trend is likely attributed to the increased flexibility influenced by an increase in the length of the aliphatic chains. Conversely, P(3,4-FMF-*co*-2,4-HF) possesses a *T*_g_ at 12 °C without a *T*_m_, suggesting the characteristics of an amorphous material. The co-FPEs (*x* = 8 and 10) show no apparent *T*_c_ in the cooling scan, possibly due to their slow crystallization kinetics. However, when utilizing an aliphatic diol chain of *x* = 12, we observed *T*_m_ during the first and second heating cycles, with *T*_c_ appearing during cooling. This observation aligns with prior research on semicrystalline polyesters derived from 3,4-DMFDCA.^32^ To validate these findings further, we conducted WAXD measurements, as discussed in the subsequent section.

### Crystallinity of Co-FPEs

**Figure 6:**
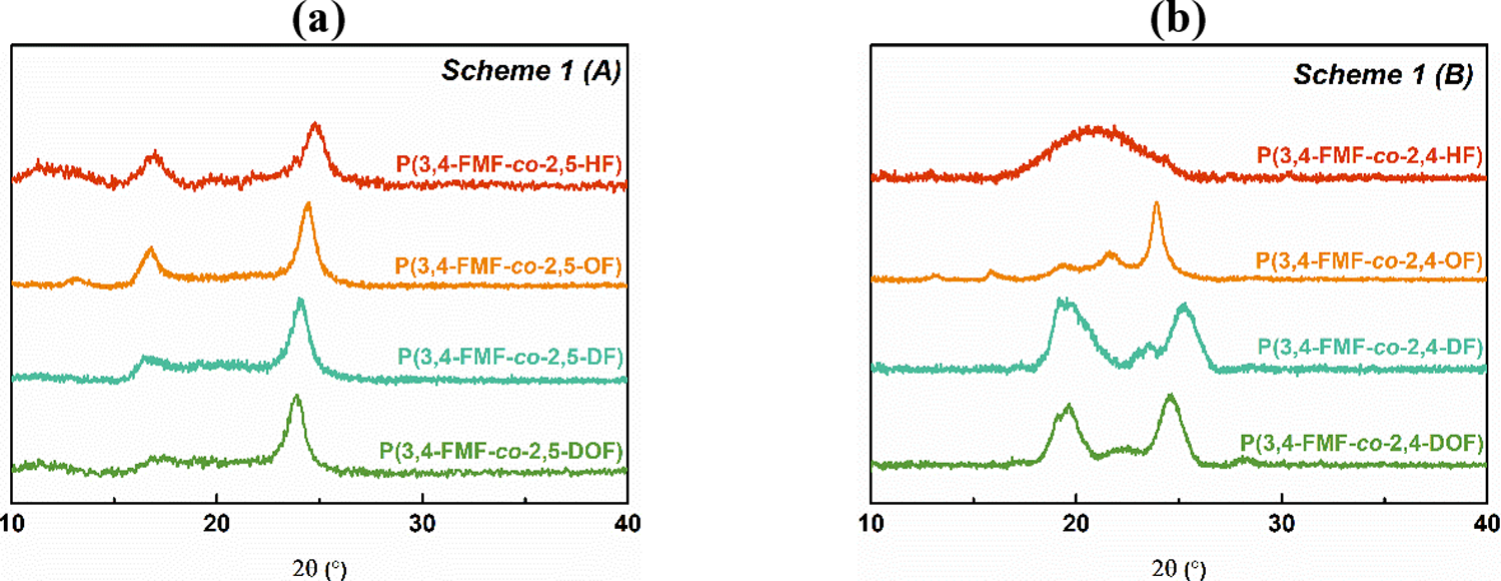
WAXD spectra of the obtained copolyesters of (a) 2,5-FDCA-based co-FPEs and (b) 2,4-FDCA-based co-FPEs.

The findings from the DSC analysis were corroborated by the WAXD patterns, further confirming the semicrystalline nature of the 2,5-FDCA-based co-FPEs. However, WAXD analysis indicated the presence of one amorphous product in the 2,4-FDCA-based co-FPEs, i.e., P(3,4-FMF-*co*-2,4-HF) (see Figure 6b). Across the 2,5-FDCA-based co-FPE WAXD spectra, a consistent diffraction pattern emerged, marked by a strong peak ranging from 22.57° to 26.23° and a smaller peak at approximately 15.30° to 18.51°, along with a broad signal extending from 9.96° to 14.05° (see Figure 6a). These observations closely align with the findings of our recent study on furanic–aliphatic copolyesters synthesized from 2,5-FDCA isomers with 2,5-BHMF through two-stage polycondensations.^4^ However, these results differ from those of reported furanic–aliphatic polyesters produced from DMA and 3,4-BHMF, which exhibit six intense peak signals from 20° to 35°.^5^ Hence, it is hypothetically proposed that the crystal structures of 2,5-FDCA-based co-FPEs more closely resemble those of their 2,5-FDCA-based counterparts than those of their 3,4-BHMF-based polyester counterparts. The diffraction peaks show degrees of crystallinity (χ_c_) ranging from 46 to 38, aligning with the trends in the *T*_m_ values.

2,4-FDCA-based co-FPEs exhibit different diffraction profiles, as shown in Figure 6b. Consistent with the DSC findings, P(3,4-FMF-*co*-2,4-HF) displays an amorphous characteristic, as evident from the broad halo diffraction in the WAXD spectra. Conversely, the 2,4-FDCA-based co-FPEs with *x* = 8–12 demonstrate semicrystalline properties with varying diffraction patterns, possibly attributed to distinct crystal packing. Notably, P(3,4-FMF-*co*-2,4-OF) displays a sharp peak at 23.89°, accompanied by four broad peaks ranging from 21.61° to 13.02°. Both P(3,4-FMF-*co*-2,4-DF) and P(3,4-FMF-*co*-2,4-DOF) exhibited three diffraction peaks within the range of 17.69° to 27.16°. Interestingly, these findings align with prior research on aliphatic polyesters based on 2,4-FDCA.^32^ Similarly, three diffraction peaks from 15° to 30° were observed in the WAXD spectrum of poly(dodecamethylene-2,4-furanoate), while poly(octamethylene-2,4-furanoate) displayed a sharp peak that was accompanied by four broad peaks.

The variation in the WAXD diffraction profiles suggested a difference in the crystallinity of the products obtained from the two DMFDCA isomers. The semicrystalline profile of the 2,5-FDCA-based co-FPEs is expected due to the symmetrical structure of 2,5-FDCA, which facilitates the arrangement of crystalline packing.^60,61^ Conversely, the asymmetrical structure of 2,4-FDCA introduces a random distribution of monomer orientations,^19^ leading to disruption of the crystal packing. Therefore, it is reasonable to conclude that the orientation of the heteroaromatic unit predominantly influences the polymer crystallinity.

## Conclusions

Furan dimethyl ester isomers (2,5- and 2,4-DMFDCA), in combination with 3,4-BHMF diol and linear aliphatic diols, were successfully employed to synthesize various co-FPEs via enzymatic polymerization. The findings revealed an enzyme preference for 2,4-DMFDCA over the 2,5-DMFDCA isomer, as indicated by the highest DP achieved, i.e., DP_*w*_ 49 and DP_*n*_ 34 for P(3,4-FMF-*co*-2,4-HF). The steric hindrance arises from the propensity of two symmetrical furanic monomers to cluster when carboxylic and hydroxymethyl groups are positioned in a reverse arrangement, while the formation of cyclic products elucidates the catalytic preference of CALB in this process. As expected, the flexible spacer, the aliphatic diol, alleviates steric hindrance, promoting a more streamlined copolymerization process. By employment of a vacuum, cyclization becomes evident in the enzymatic polymerization of the symmetrical 2,5-DMFDCA isomer. The opposite outcome was observed for the asymmetrical counterpart 2,4-DMFDCA. Comprehensive material analysis revealed that isomeric switching within the co-FPE structure resulted in unique thermal characteristics, confirming that copolyester degradation comprises two distinct steps, i.e., a heteroaromatic unit and an aliphatic unit. Nearly identical *T*_g_ values of 9 and 10 °C were observed for all the obtained 2,5-FDCA-based co-FPEs. Additionally, all 2,4-FDCA-based co-FPEs possess a *T*_g_ below 0 °C. The WAXD results align with the DSC analysis and verify the semicrystalline nature of all 2,5-FDCA-based co-FPEs, while 2,4-FDCA-based co-FPEs showed varied diffraction profiles, and amorphous materials were discovered for P(3,4-FMF-*co*-2,4-HF). This can be explained by considering the impact of the orientation of the heteroaromatic unit and the methylene aliphatic unit. These findings hold the potential for further exploration of enzymatic polymerization based on isomeric variation of furan-based polymers. Furthermore, elucidating the properties of these furanic isomeric co-FPEs provides valuable insight into their prospective applications. For future research, exploring the variation in the monomeric feed of isomeric furanic esters and 3,4-BHMF, as well as the variation in polymerization conditions, would provide further insights.

## Supporting Information Available

Characterization data (FTIR and ^1^H NMR), data analysis from MALDI-ToF results, table of molecular weights, dispersities, yield, and material properties (PDF)
