# Supplementary material for: Enzymatic Synthesis of Copolyesters with the Heteroaromatic Diol 3,4-Bis(hydroxymethyl)furan and Isomeric Dimethyl Furandicarboxylate Substitutions
Source: Biomacromolecules. 2024 Apr 11;25(5):2792–802. doi: 10.1021/acs.biomac.3c01433 (PMC11094730; doi:10.1021/acs.biomac.3c01433)
Supplement: Supplementary file 1 — bm3c01433_si_001.pdf [file bm3c01433_si_001.pdf]

Supplementary Information

Enzymatic synthesis of copolyesters with the hetero-aromatic diol 3,4-bis(hydroxymethyl)furan and isomeric dimethyl furandicarboxylate substitutions

Fitrilia Silvianti [1], Dina Maniar [1], Beatriz Agostinho [2], Tijn C. de Leeuw [3], Albert Jan Jacob Woortman [1], Jur van Dijken [1], Shanmugam Thiyagarajan [5], Andreia F. Sousa [2,4], and Katja Loos [1,*]

1 Macromolecular Chemistry & New Polymeric Materials, Zernike Institute for Advanced Materials, University of Groningen, Nijenborgh 4, 9747 AG, Groningen, The Netherlands

2 CICECO—Aveiro Institute of Materials, Department of Chemistry, University of Aveiro, 3810-193 Aveiro, Portuga

3 CarbExplore Research B.V., 9747 AA Groningen, The Netherlands

4 Centre for Mechanical Engineering, Materials and Processes, Department of Chemical Engineering, University of Coimbra Rua Sílvio Lima – Polo II, 3030-790 Coimbra, Portugal

5 Wageningen Food & Biobased Research, Wageningen University and Research, Wageningen, P. O. Box 17, 6700 AA, The Netherlands

* Corresponding Author: k.u.loos@rug.nl

(a)

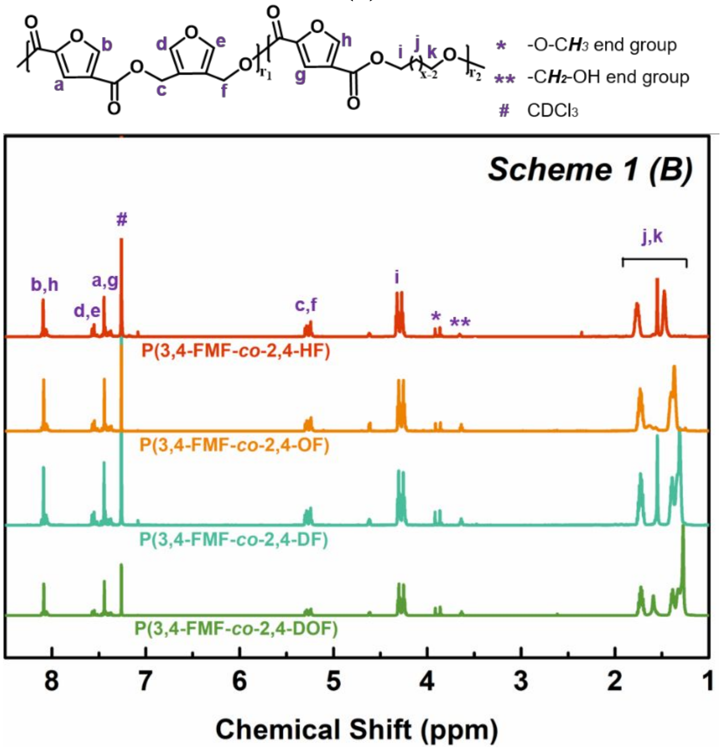

(b)

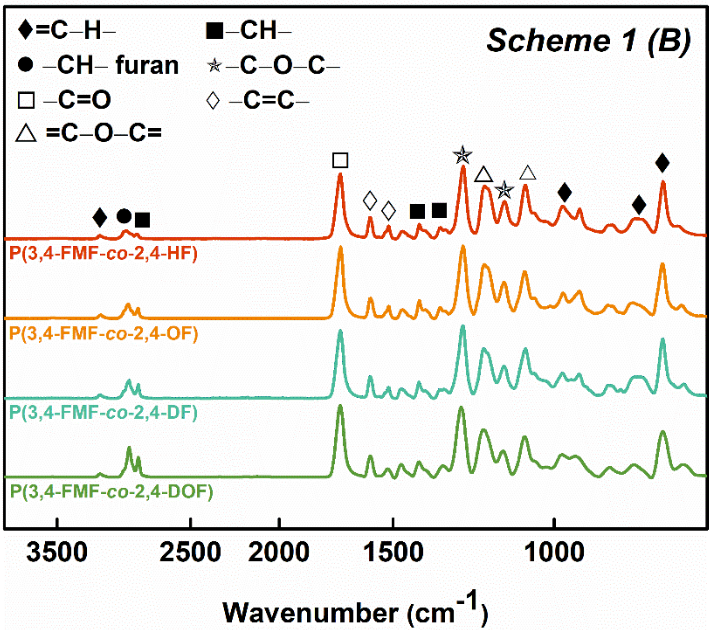

Figure S1. (a) ^{1}H-NMR and (b) ATR-FTIR spectra of co-FPEs obtained from enzymatic polymerization of 3,4-BHMF, 2,4-DMFDCA, and aliphatic diols.

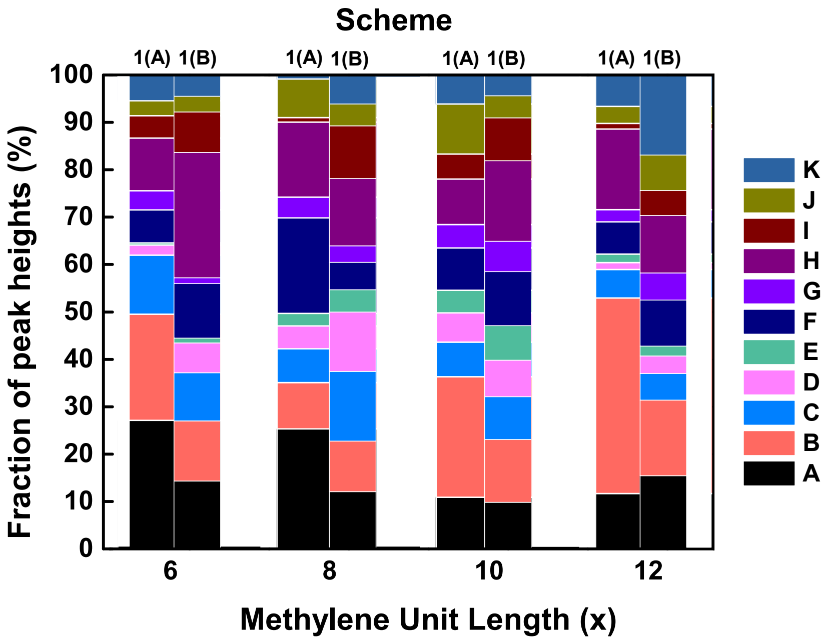

Figure S2. The sum of peak height intensities of co-FPEs product species from MALDI-ToF MS analysis; microstructure of species A to K are provided in Table 2.

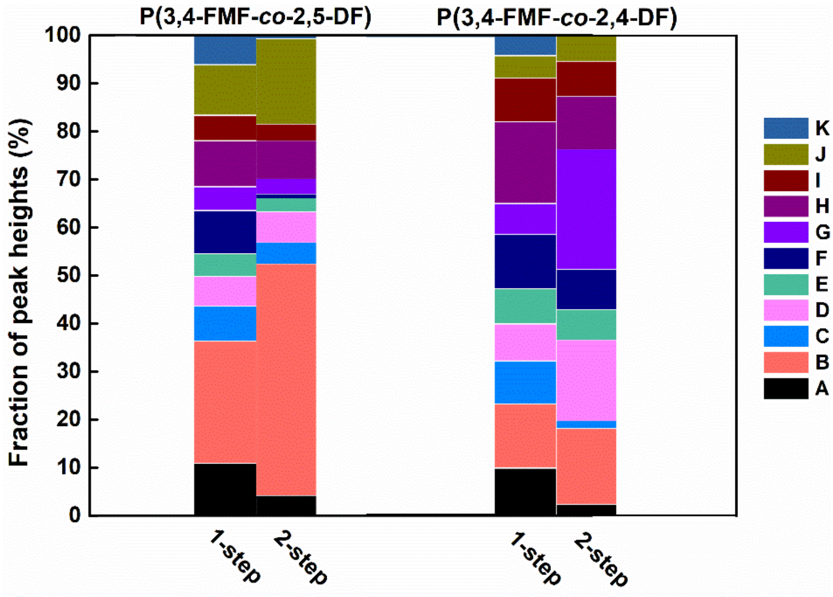

Figure S3. Comparison of the sum of peak height intensities of product species from MALDI-ToF MS analysis of P(3,4-FMF-*co*-2,5-DF) and P(3,4-FMF-*co*-2,4-DF); microstructure of species A to K are provided in Table 2.

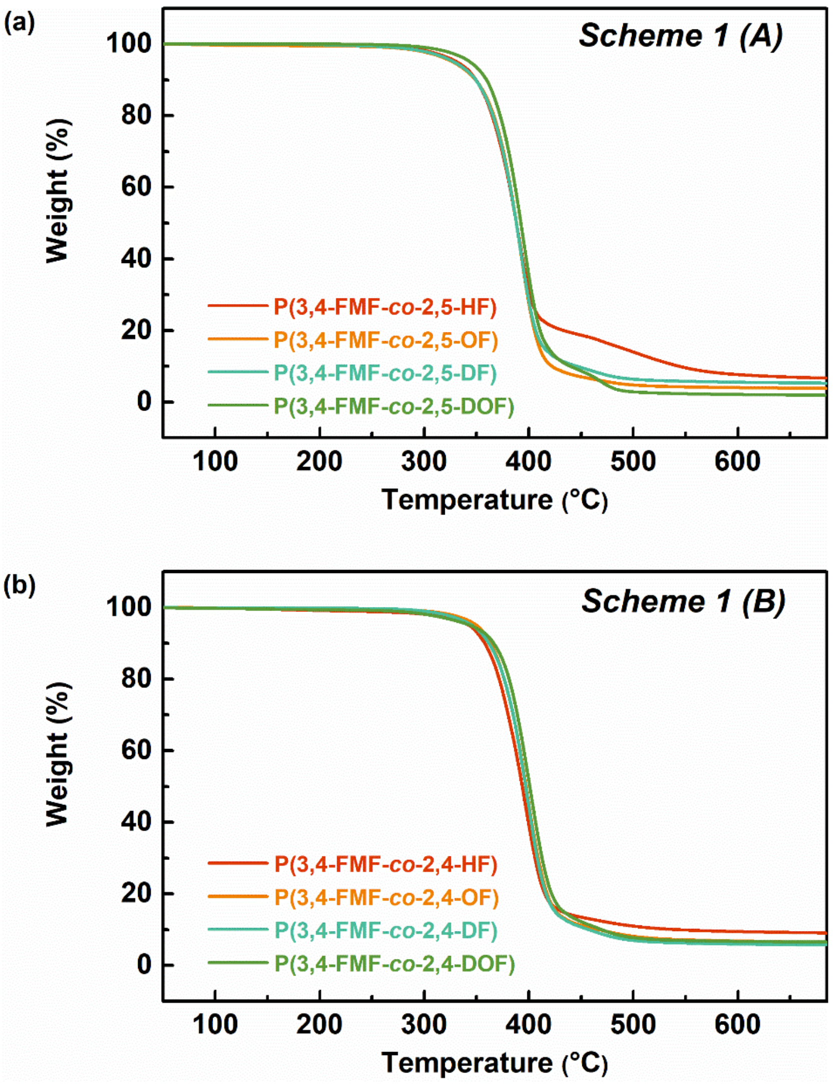

Figure S4. TGA curves of the obtained copolyesters from (a) 2,5-DMFDCA, 3,4-BHMF, and aliphatic diols (b) 2,4-DMFDCA, 3,4-BHMF, and aliphatic diols.

NMR.

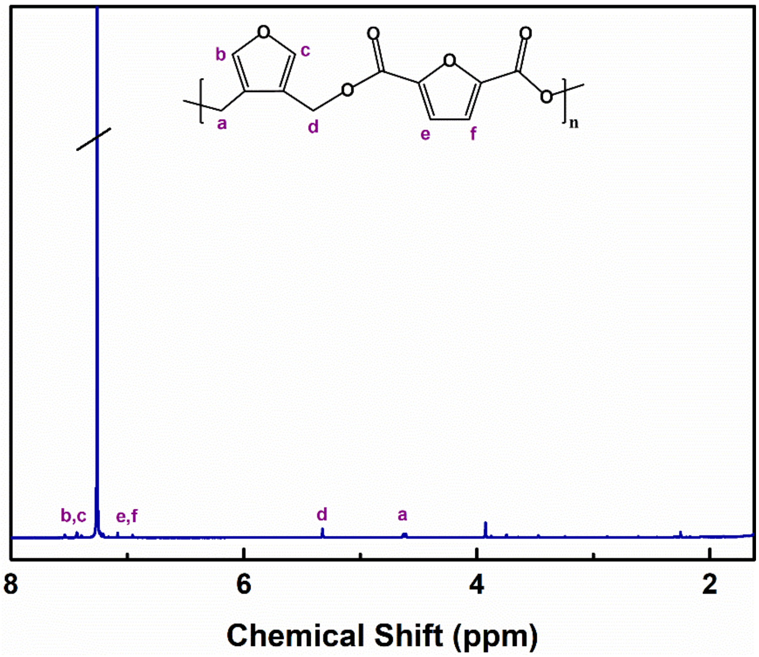

Figure S5. ^{1}H-NMR spectra of product obtained from enzymatic polymerization of 3,4-BHMF and 2,5-DMFDCA

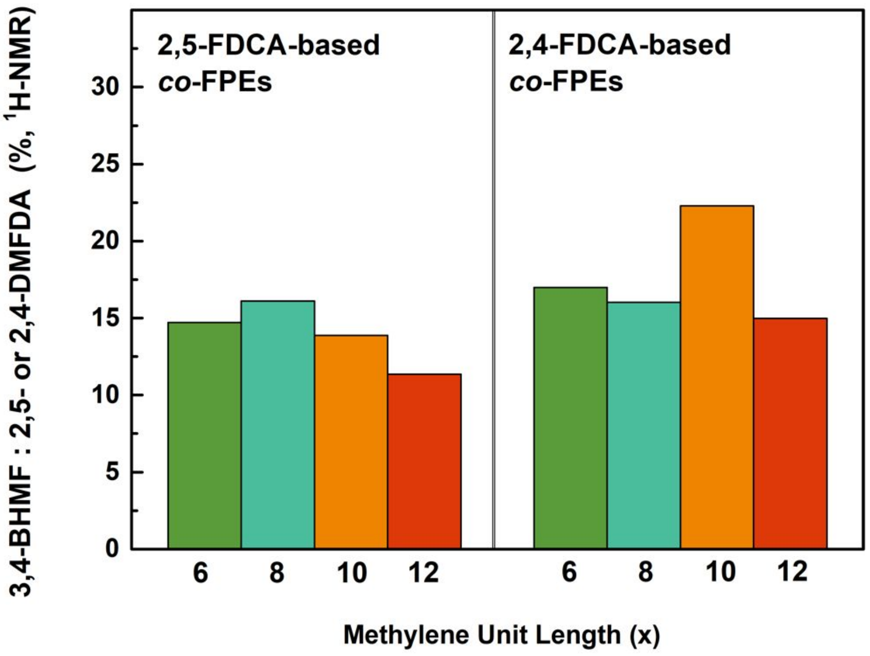

Figure S6. The molar fraction percentage of 3,4-BHMF incorporated in the obtained 2,5- and 2,4-based co-FPEs with various methylene unit length of aliphatic diols determined by ^{1}H-NMR.

Table S1. Molecular weights, dispersities, and yields of the obtained furan-based copolyesters

x[a]	DMFDCA Isomers	Copolyester	$\overline{M_n}$[b] [g mol^{-1}]	$\overline{M_w}$[b] [g mol^{-1}]	Đ[b] ($\overline{M_w}/\overline{M_n}$)	Yield[c] [%]
6		P(3,4-FMF-*co*-2,5-HF)	2900	4700	1.6	28
8	2,5-DMFDCA	P(3,4-FMF-*co*-2,5-OF)	3200	5100	1.6	35
10		P(3,4-FMF-*co*-2,5-DF)	3550	5700	1.6	27
12		P(3,4-FMF-*co*-2,5-DOF)	4200	7100	1.4	26
6		P(3,4-FMF-*co*-2,4-HF)	4100	5900	1.5	36
8	2,4-DMFDCA	P(3,4-FMF-*co*-2,4-OF)	4400	6000	1.4	29
10		P(3,4-FMF-*co*-2,4-DF)	4000	6500	1.9	53
12		P(3,4-FMF-*co*-2,4-DOF)	4600	7100	1.5	42

[a] The number of methylene units in aliphatic linear diols, [b] The number-average molecular weight ($\overline{M_n}$), weight-average molecular weight ($\overline{M_w}$), and dispersity (Đ, $\overline{M_w}/\overline{M_n}$) were determined by SEC using $CHCl_3$ as the eluent; [c] isolated yield.

Table S2. Thermal and crystalline properties of the obtained furan-based copolyesters

DMFDCA Isomers	Copolyester	DSC					TGA[e]	WAXD[f]
		$T_g^{a,c}$ (°C)	T_m^b (°C)	T_m^c (°C)	T_{cc}^c (°C)	T_c^d (°C)	$T_{d\text{-}max}$ (°C)	χ_c
2,5-DMFDCA	P(3,4-FMF-*co*-2,5-HF)	10	57; 122	108; 123	-[g]	79	389; 501	43
	P(3,4-FMF-*co*-2,5-OF)	10	104; 123	112; 124	-[g]	94	389; 478	46
	P(3,4-FMF-*co*-2,5-DF)	9	69; 97	95	-[g]	75	391; 469	38
	P(3,4-FMF-*co*-2,5-DOF)	10	53; 95	95	-[g]	75	394; 467	38
2,4-DMFDCA	P(3,4-FMF-*co*-2,4-HF)	12	-[g]	-[g]	-[g]	-[g]	396; 476	-[g]
	P(3,4-FMF-*co*-2,4-OF)	-2	43; 59	-[g]	-[g]	-[g]	398; 480	36
	P(3,4-FMF-*co*-2,4-DF)	-7	51	-[g]	-[g]	-[g]	397; 474	37
	P(3,4-FMF-*co*-2,4-DOF)	-14	44; 61	46; 54	26	-[g]	400; 476	35

[a] T_g = glass transition temperature of the 2,5-FDCA-based co-FPEs analyzed from the modulated DSC heating scan, [b] T_m = melting temperature analyzed from the first heating scan, [c] T_g of the 2,4-FDCA-based co-FPEs , T_m, and T_{cc} = cold crystallization temperature analyzed from the second heating scan, [d] T_c = crystallization temperature from the cooling scan; [e] $T_{d\text{-}max}$ = temperature at the maximum rate of decomposition; [f] The degree of crystallinity (χ_c) was calculated from WAXD; [g] not determined.

Furan-based copolyesters

ATR-FTIR (ν, cm^{-1}): 3118 - 3137 (=C-H stretching vibration of the furan ring); 2780 - 3021 (C-H stretching vibrations); 1720 - 1724 (C=O stretching vibration of ester); 1573 – 1583, 1506–1511 (aromatic C=C bending vibrations); 1443 - 1471, 1371 - 1392 (C-H deformation and wagging vibration); 1329 (C-H rocking vibration); 1122 - 1151, 1268 - 1276 (asymmetrical and symmetrical stretching vibrations of the ester C-O-C group); 1203 - 1228, 1073 - 1079, 1010 - 1016 (=C-O-C= ring vibration, furan ring); 948 - 979, 820 - 822, 760 (=C-H out-of-plane deformation vibration, furan ring).

Poly(3,4-furandimethylene furanoate-co-hexamethylene-2,5-furanoate)

^{1}H-NMR (600 MHz, $CDCl_3$, δ, ppm): 7.18 (2H, s, –C**H**=, DMFDCA); 7.56 (2 H, s, -C**H**=, BHMF); 5.32 (4 H, s, -CO-O-C**H**$_2$-, BHMF); 4.33 (4H, m, –CO–O–C**H₂**–, from 1,6-HDO); 1.78 (4H, m, –CO–O–CH_2–C**H**$_2$–, from 1,6-HDO); 1.47 (4H, m, –C**H**$_2$–, from 1,6-HDO); 3.92 (s, –O–C**H**$_3$, end group from DMFDCA); 3.65 (t, –C**H**$_2$–OH, end group from 1,6-HDO); and 4.62 (s, -C**H**$_2$OH, end group from BHMF).

Poly(3,4-furandimethylene furanoate-co-hexamethylene-2,4-furanoate)

^{1}H-NMR (600 MHz, $CDCl_3$, δ, ppm): 7.44; 8.09 (2H, s, –C**H**=, DMFDCA), 7.54; 7.57 (2 H, s, -C**H**=, BHMF), 5.24; 5.25; 5.28; 5.30 (4 H, s, -CO-O-C**H**$_2$-, BHMF), 4.27; 4.32 (4H, m, –CO–O–C**H**$_2$–, from 1,6-HDO), 1.76 (4H, m, –CO–O–CH_2–C**H**$_2$–, from 1,6-HDO), 1.47 (4H, m, –C**H**$_2$–, from 1,6-HDO), 3.86; 3.92 (s, –O–C**H**$_3$, end group from DMFDCA), 3.65 (t, –C**H**$_2$–OH, end group from 1,6-HDO), 4.62 (s, -C**H**$_2$OH, end group from BHMF).

Poly(3,4-furandimethylene furanoate-co-octamethylene-2,5-furanoate)

^{1}H-NMR (600 MHz, $CDCl_3$, δ, ppm): 7.18 (2H, s, –C**H**=, DMFDCA), 7.56 (2 H, s, -C**H**=, BHMF), 5.31 (4 H, s, -CO-O-C**H**$_2$-, BHMF), 4.31 (4H, m, –CO–O–C**H**$_2$–, from 1,8-ODO), 1.75 (4H, m, –CO–O–CH_2–C**H**$_2$–, from 1,8-ODO), 1.39 (4H, m, –C**H**$_2$–, from 1,8-ODO), 3.92 (s, –O–C**H**$_3$, end group from DMFDCA), 3.65 (t, –C**H**$_2$–OH, end group from 1,8-ODO), 4.62 (s, -C**H**$_2$OH, end group from BHMF).

Poly(3,4-furandimethylene furanoate-co-octamethylene-2,4-furanoate)

^{1}H-NMR (600 MHz, $CDCl_3$, δ, ppm): 7.44; 8.09 (2H, s, –C**H**=, DMFDCA); 7.54; 7.56 (2 H, s, -C**H**=, BHMF); 5.24; 5.25; 5.28; 5.30 (4 H, s, -CO-O-C**H**$_2$-, BHMF); 4.25; 4.30 (4H, m, –CO–O–C**H**$_2$–, from 1,8-ODO); 1.76 (4H, m, –CO–O–CH_2–C**H**$_2$–, from 1,8-ODO); 1.47 (4H, m, –C**H**$_2$–, from 1,8-ODO); 3.86; 3.91 (s, –O–C**H**$_3$, end group from DMFDCA); 3.64 (t, –C**H**$_2$–OH, end group from 1,8-ODO); 4.62 (s, -C**H**$_2$OH, end group from BHMF).

Poly(3,4-furandimethylene furanoate-co-decamethylene-2,5-furanoate)

^{1}H-NMR (600 MHz, $CDCl_3$, δ, ppm): 7.18 (2H, s, –C**H**=, DMFDCA), 7.56 (2 H, s, -C**H**=, BHMF), 5.31 (4 H, s, -CO-O-C**H**$_2$-, BHMF), 4.31 (4H, m, –CO–O–C**H**$_2$–, from 1,10-DDO), 1.73 (4H, m, –CO–O–CH_2–C**H**$_2$–, from 1,8-ODO), 1.39 (4H, m, –C**H**$_2$–, from 1,10-DDO), 3.92 (s, –O–C**H**$_3$, end group from DMFDCA), 3.63 (t, –C**H**$_2$–OH, end group from 1,10-DDO), 4.62 (s, -C**H**$_2$OH, end group from BHMF)

Poly(3,4-furandimethylene furanoate-co-decamethylene-2,4-furanoate)

^{1}H-NMR (600 MHz, $CDCl_3$, δ, ppm): 7.44; 8.08 (2H, s, –C**H**=, DMFDCA); 7.55; 7.57 (2 H, s, -C**H**=, BHMF); 5.24; 5.25; 5.28; 5.30 (4 H, s, -CO-O-C**H**$_2$-, BHMF); 4.25; 4.30 (4H,

m, –CO–O–C**H₂**–, from 1,10-DDO); 1.72 (4H, m, –CO–O–CH_2–C$\mathbf{H}_2$–, from 1,10-DDO); 1.39 (4H, m, –C$\mathbf{H}_2$–, from 1,10-DDO); 3.86; 3.91 (s, –O–C$\mathbf{H}_3$, end group from DMFDCA); 3.64 (t, –C$\mathbf{H}_2$–OH, end group from 1,10-DDO); 4.61 (s, -C$\mathbf{H_2}$OH, end group from BHMF).

Poly(3,4-furandimethylene furanoate-co-dodecamethylene-2,5-furanoate)

^{1}H-NMR (600 MHz, $CDCl_3$, δ, ppm): 7.18 (2H, s, –C**H**=, DMFDCA), 7.56 (2 H, s, -C**H**=, BHMF), 5.32 (4 H, s, -CO-O-C$\mathbf{H}_2$-, BHMF), 4.31 (4H, m, –CO–O–C$\mathbf{H_2}$–, from 1,12-DODO), 1.74 (4H, m, –CO–O–CH_2–C$\mathbf{H}_2$–, from 1,12-DODO), 1.39 (4H, m, –C$\mathbf{H}_2$–, from 1,12-DODO), 3.92 (s, –O–C$\mathbf{H}_3$, end group from DMFDCA), 3.63 (t, –C$\mathbf{H}_2$–OH, end group from 1,12-DODO), 4.63 (s, -C$\mathbf{H_2}$OH, end group from BHMF)

Poly(3,4-furandimethylene furanoate-cododecamethylene-2,4-furanoate)

^{1}H-NMR (600 MHz, $CDCl_3$, δ, ppm): 7.44; 8.08 (2H, s, –C**H**=, DMFDCA); 7.54; 7.56 (2 H, s, -C**H**=, BHMF); 5.24; 5.25; 5.28; 5.30 (4H, s, -CO-O-C$\mathbf{H}_2$-, BHMF); 4.25; 4.30 (4H, m, –CO–O–C$\mathbf{H_2}$–, from 1,12-DODO); 1.72 (4H, m, –CO–O–CH_2–C$\mathbf{H}_2$–, from 1,12-DODO); 1.39 (4H, m, –C$\mathbf{H}_2$–, from 1,12-DODO); 3.86; 3.91 (s, –O–C$\mathbf{H}_3$, end group from DMFDCA); 3.64 (t, –C$\mathbf{H}_2$–OH, end group from 1,12-DODO); 4.61 (s, -C$\mathbf{H_2}$OH, end group from BHMF)
